# Living donor renal transplantation in patients with antiphospholipid syndrome: A case report

**DOI:** 10.1097/MD.0000000000005419

**Published:** 2016-11-18

**Authors:** Ji Yoon Choi, Joo Hee Jung, Sung Shin, Young Hoon Kim, Duck Jong Han

**Affiliations:** Department of Surgery, University of Ulsan College of Medicine and Asan Medical Center, Seoul, Korea.

**Keywords:** antiphospholipid antibody syndrome, graft outcome, renal transplantation

## Abstract

**Introduction:**

Antiphospholipid syndrome (APS), autoantibodies directed against phospholipid-binding proteins are associated with cause vascular thrombosis. Patients with APS requiring renal transplantation are at risk of early graft loss due to arterial or venous thrombosis, or thrombotic microangiopathy (TMA). Here, we report 3 cases of successful renal transplantation in patients with APS.

**Clinical Findings:**

A 53-year-old man with end-stage renal disease (ESRD) had experienced bilateral deep venous thrombosis (DVT) in the lower extremities 16 years ago and was administered warfarin. However, he frequently experienced recurrent DVT despite of anticoagulation therapy. Before the surgery, APS was confirmed based on positive results lupus anticoagulant in serological tests. A 40-year-old man with polycystic kidney disease and a history recurrent DVT tested positive for lupus anticoagulant and anticardiolipin antibodies. Lastly, a 42-year-old woman with ESRD was diagnosed with APS 7 years ago. She also developed DVT and tested positive for lupus anticoagulant and anti-B2-glycoprotein 1.

**The anticoagulation protocol was as follows in all cases:**

Warfarin was stopped 5 days before living donor renal transplantation and intravenous heparin therapy was started. During surgery, bolus heparin injections (3000 U) were administered to prevent arterial or venous thrombosis. Heparin was substituted with warfarin on postoperative day 4. The third patient (42/F) developed clinical rejection indicated by increased serum creatinine levels and donor-specific antibodies (DSA) and received steroid pulse therapy, plasmapheresis, and rituximab. This treatment restored graft function to within the normal range. The latest graft function in all patients was maintained at normal levels in the outpatient clinic.

**Conclusions:**

Living donor renal transplantation may be successful in patients with APS following perioperative anticoagulation therapy. However, because of the high risk of TMA or vascular thrombosis in the early postoperative period, close monitoring for hypercoagulability and continuous anticoagulation is essential for maintaining graft function.

## 1 Introduction

Antiphospholipid syndrome (APS) is an autoimmune disorder in which autoantibodies are directed against phospholipid-binding proteins. It is clinically characterized by recurrent arterial and/or venous thrombosis (often multiple thrombosis), and frequent fetal loss, often accompanied by moderate thrombocytopenia.^[1–5]^ It is diagnosed by the presence of antiphospholipid antibodies (aPL), including lupus anticoagulant (LA), anticardiolipin (aCL), and anti-β2-glycoprotein I (anti-β2GPI) antibodies in the plasma and must be confirmed by repeated testing 12 weeks later.^[1,4,5]^

The hypercoagulable state potentially resulting in thrombosis can develop in all segments of the vascular bed and solid organs, such as the liver, pancreas, spleen, intestine, and kidney. Occasionally, APS may cause thrombotic microangiopathy (TMA), involving microvascular endothelial injury, intimal expansion and fibrin deposition culminating in microvascular thrombosis. To prevent recurrent arterial and/or venous thrombosis, anticoagulation therapy is recommended for patients with APS ^[1,4,6]^ Growing evidence suggests that patients with end-stage renal disease (ESRD) and APS are at a high risk for renal vascular thrombosis, graft failure, and/or systemic thrombosis after renal transplantation.^[1–3,7]^ Although anticoagulation therapy before and at the time of renal transplantation can reduce the risk of early post-transplant thrombosis, allograft thrombosis can develop despite this treatment. Moreover, it increases the risk of bleeding complications, which can lead to early allograft loss. Only a few studies have examined the early post-transplant outcomes of renal transplant recipients with APS.^[1–3]^

Therefore, we reviewed the cases of 3 patients with APS who underwent renal transplantation at our center. This study was approved by the Institutional Review Board of the AMC (2014-0776).

## 2 Case presentation

The first case is that of a 53-year-old man who was referred to our renal transplantation center. He had been diagnosed with hypertension and diabetes mellitus 17 years ago and had experienced hematuria and proteinuria 16 years ago. He had consulted a nephrologist, and kidney biopsy had revealed focal segmental glomerular sclerosis (FSGS). His renal function remained relatively good for several years, but started deteriorating gradually. He had started hemodialysis 2 months before visiting our center. Examination of his medical history also showed that he had experienced edema in both legs 16 years ago. Duplex ultrasonography of the lower extremities at that time had shown deep venous thrombosis (DVT) throughout the length of the superficial femoral, popliteal, and calf veins. The patient had been started on anticoagulation therapy with warfarin to maintain the prothrombin (PT) time between 2.0 and 3.0 international normalized ratio (INR). Despite this, 7 years and 1 year ago, he experienced recurrent bilateral DVT that was confirmed by computed tomographic angiography (CTA), the patient tested positive for LA antibodies and was confirmed to have APS.

**Figure 1:**
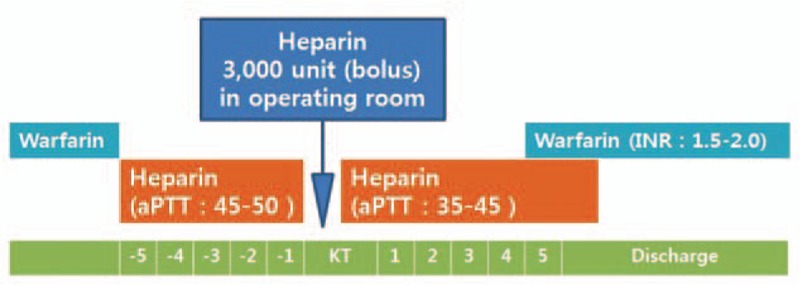
Anticoagulation protocol of recipients with antiphospholipid antibody syndrome in perioperative periods. Our center changed anticoagulation from warfarin to i.v. heparin before surgery and maintained until postoperative 4 to 5 day.

At our center, renal transplantation was planned with the patient's son as the donor, and the patient underwent plasmapheresis to prevent recurrence of FSGS in the early postoperative period. The immunosuppressive regimen was a combination of tacrolimus, mycophenolate mofetil (1500 mg/day), and methylpredinisolone, and induction with basiliximab. Anticoagulation therapy with warfarin (3 mg/day) was replaced with daily continuous injection of heparin, starting 5 days before transplantation. The dose of heparin was adjusted to maintain an activated partial thromboplastin time (aPTT) of 40 to 60 seconds, and the injection was stopped 6 hours before surgery. During the surgery, we administered a bolus heparin injection (3000U) in order to prevent arterial or venous thrombosis before vascular clamping. Continuous heparin injection was restarted in the evening of the day of surgery, with the dose adjusted to maintain an aPTT of 35 to 45, and it was replaced on day 4 by warfarin at the dose to maintain the INR at 1.5 to 2.0 (Fig. 1).

**Figure 2:**
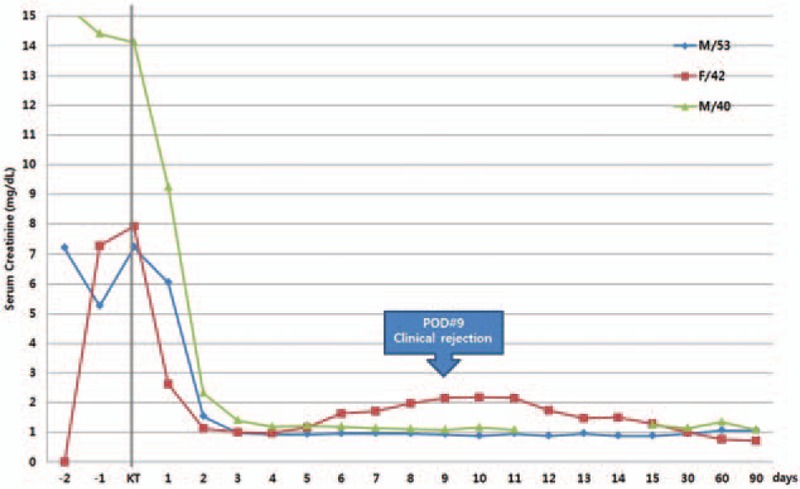
Renal allograft function as measured by serum creatinine within 100 days of renal transplantation. Serum creatinine of all patients was normal during the follow-up periods in outpatient clinic.

After transplantation, there were no obvious thrombotic or bleeding complications. The patient was discharged 16 days after transplantation, with a prescription for anticoagulation therapy with warfarin (4 mg/day). His serum creatinine level was 0.87 mg/dL and remained stable at 4 months after discharge (0.96 mg/dL) (Fig. 2).

The second case is that of a 40-year-old man with polycystic kidney disease who was scheduled for renal transplantation with his sister as a donor. Four years earlier, he developed dyspnea and atypical chest pain. Computed tomography of the chest and lower extremities revealed pulmonary thromboembolism of both central pulmonary arteries and DVT of the left popliteal vein. Anticoagulation therapy with warfarin (6–7 mg/day) was started and maintained. A lung perfusion scan conducted before the surgery and showed small to medium sized ventilation/perfusion(V/Q) mismatching defects that could lead to pulmonary embolism(PE) in both upper and lower lobes. Serologic testing showed LA and aCL IgM positivity and APS was diagnosed by a pulmonologist. Five days before transplantation, warfarin, was replaced with continuous heparin injection, which was stopped 6 hours before surgery. During the surgery, low-molecular-weight heparin (20 IU/kg) was administered to prevent thrombosis. Heparin was restarted on postoperative day 1 and replaced with warfarin on day 4 (Fig. 1). After transplantation, the serum creatinine level remained stable (1.08 mg/dL) and the patient was discharged on postoperative day 11 with on warfarin therapy (7 mg/day). Three months after discharge, his renal function was stable without any complications (Fig. 2).

In the third case, a 42-year-old woman with ESRD was diagnosed with APS 7 years earlier. She experienced edmea, paresthesia, and coldness in the left leg, and CT angiography, showed popliteal artery occlusion. The patient tested positive for LA and anti-B2-glycoprotein 1 antibodies on serological examination. She was then started on anticoagulation therapy. The level of calculated panel reactive antibody (PRA) was high (class I: 44%, class II; 60%) and donor-specific antibodies (DSAs) were detected with a relatively low mean fluorescence intensity (MFI) (HLA-DQ7; 1598). Five days before surgery, warfarin was replaced with continuous heparin injection. During the surgery, a bolus injection of intravenous heparin (3000U) was administered to prevent vascular thrombosis. Continuous heparin injection was restarted in the evening of the day of surgery and adjusted to maintain an aPTT of 45 to 50 seconds (Fig. 1). Five days after the operation, the patient developed severe lower abdominal pain and her serum hemoglobin levels decreased from 9.3 to 7.5 mg/dL. Abdominal computed tomography (CT) was performed to rule out retroperitoneal bleeding, and a 6 × 7 cm hematoma was detected. Further, her serum creatinine levels increased from 0.97 to 1.63 mg/dL. Exploratory laparotomy was done and there was a large hematoma near the uretero-neocystostomy site. The arterial and venous anastomosis sites were intact. The hematoma was evacuated on postoperative day 6. After this operation, we restarted anticoagulation therapy with heparin to prevent vascular thrombosis, but, we lowered the target range of aPTT (35–40 seconds). Her serum creatinine level increased to 2.16 mg/dL and repeated DSA testing revealed the development of de novo DSAs and increased MFI of the existing DSAs. Although renal biopsy is needed to confirm graft rejection, we diagnosed graft rejection clinically on the bases of the increased MFI of the DSA, de novo DSA formation after transplantation, and elevated serum creatinine level, since the patient was receiving anticoagulation therapy. Despite being administered steroid pulse therapy (2.5 g), the MFI of the existing DSAs increased further and another de novo DSA was detected. Therefore, we performed plasmapheresis 4 times and administered rituximab therapy (200 mg) for antibody mediated rejection. After the treatment, the serum creatinine levels reduced to 1.10 mg/dL. Follow-up abdominal CT showed the persistence of a small hematoma and some fluid collection. The patient was discharged with a surgical drainage tube on day 24 after renal transplantation. Abdominal CT conducted in the outpatient clinic showed increased fluid collection around the transplanted kidney suggesting the complicated fluid originated from remnant hematoma and lymphocele. We inserted another drainage tube, which was kept for 1 week and removed after the resolution of fluid collection was confirmed by CT. The patient's serum creatinine level remained stable (0.90 mg/dL) (Fig. 2) at 4 months after discharge.

## 3 Discussion

It is well known that patients with ESRD and APS are at a relatively high risk for renal vascular thrombosis and/or graft failure after renal transplantation.^[1–3,7]^ Most patients are diagnosed with APS on the basis of presence of recurrent DVT or pulmonary embolism (PE), and positive results in serial tests for LA and/or high-titer aCL.^[1,5]^

Giannakopoulos and Krilis^[8]^ first described the “two hit” model of thrombosis in which the “first hit” injury disrupts the endothelium and the “second hit” potentiates thrombus formation and TMA. “First hit” injuries include trauma, surgery, drugs, and infection, and surgical procedures are important risk factor for thrombosis in patients for APS. Therapeutic anticoagulation is recommended for all APS patients with a history of DVT, PE, or arterial thrombosis.^[4,5]^ For renal transplant recipients, low-molecular-weight or unfractionated heparin is preferred for prophylaxis of allograft thrombosis because of their short half-life.^[6]^

We encountered 3 cases of patients with APS who received renal transplant from living donors. The recipients had a history of DVT or PE, and had been diagnosed with APS via repeated serological testing. We established a protocol to prevent APS-related thrombotic events after renal transplantation. If the recipients had a history of recurrent arterial or venous thrombosis, or APS, we repeated serological tests to confirm whether they had antiphospholipid antibodies such as LA, aCL, and anti-β2GPI. If the results were positive, we consulted a rheumatologist or hematologist to confirm the diagnosis of APS. Since all recipients were already taking warfarin, we changed the anticoagulation therapy in the perioperative period (Fig. 1). In all cases, warfarin was stopped 5 days before renal transplantation and replaced with continuous heparin injection because the half-life of heparin is shorter than those of warfarin. The dose of heparin was adjusted to maintain an aPTT of 40 to 50 seconds, which is slightly lower than the target aPTT in the general treatment protocol for DVT or PE, and heparin was discontinued 6 hours before surgery. During surgery, bolus heparin (3000 U) were administered just before vascular clamping to prevent arterial or venous thrombosis by clamping or reperfusion. No perioperative thrombotic complications occurred. Continuous heparin injection was restarted about 6 to 8 hours after surgery, with the dose adjusted to maintain an aPTT of 35 to 45 seconds. This was switched to warfarin on day 4 to maintain an INR of 1.5 to 2.0 and this anticoagulation therapy was maintained after discharge.

Vaidya^[9]^ showed that graft survival in patients with APS is not influenced by the type of anticoagulation therapy used; however, the 10-year graft survival is significantly lower in recipients with APS than in those without APS. Although many studies have shown that perioperative anticoagulation therapy can reduce the rate of graft thrombosis, they have also shown a corresponding increase in bleeding complications.^[1,5,9]^ Even though we used low-dose anticoagulation therapy (aPTT, 35–45 seconds; INR, 1.5–2.0), 1 case in the present study showed postoperative bleeding. In some previous cases, bleeding has led to graft loss, whereas in others, anticoagulation therapy had to be stopped because of subsequent graft thrombosis.^[1]^

In 1 of our patients, clinical rejection developed after transplantation. Her serum creatinine levels increased, and we were not able to perform a renal biopsy because she had already undergone hematoma evacuation at this time because of postoperative bleeding. A peripheral blood smear test showed no TMA, but the kidney scan showed mildly decreased perfusion and glomerular filtration rates of the transplanted kidney, suggesting rejection. The patient already had low titer DSAs before transplantation. After surgery, the titer increased and de novo DSAs developed, as well. We believed that antibody mediated rejection had occurred because of the DSA despite its lower titer. However, it was not clear APS-related TMA had developed because we could not perform a renal biopsy to confirm TMA. Antibody-mediated rejection because of the DSA could explain the elevated serum creatinine levels, and, the possibility that APS-related TMA hampered renal function and elevated serum creatinine levels could not be completely excluded. The patient was administered steroid pulse, plasmapheresis, and rituximab treatment under a clinical diagnosis of antibody-mediated rejection, and her renal function recovered.

Many studies have shown that preoperative or post-operative plasmapheresis can help maintain the renal function of renal transplant recipients with APS.^[1–3,10]^ Sofue^[2]^ successfully performed living donor kidney transplantation in a patient with secondary APS following a combination of prophylactic plasmapheresis and anticoagulation therapy. The plasmapheresis reduced the LA, aCL, and anti-β2GPI levels to the normal ranges. The study by Barbour's group^[1]^ showed evidence of treatment of APS-related renal TMA. For APS related allograft TMA, plasmapheresis was reportedly associated with a good response and contributes to partial renal recovery. TMA was resolved following prompt treatment with daily plasmapheresis, intravenous immunoglobulin, and high-dose steroids. We, too, administered a combination of plamapheresis, high-dose steroids and rituximab, and were successful in maintaining the patient's immunity and recovering graft function.

Substantial efforts have been made recently to identify how APS affects renal allografts and to prevent allograft thrombosis.^[1,3,7,11]^ Forman et al^[7]^ analyzed the influence of aCL antibodies on short- and long-term allograft survival and function following kidney transplantation. They found no significant differences in overall graft outcomes between patients with or without a positive aCL titer, although the significance of very high titers remains to be determined. Some studies have found that the titer of an autoantibody is an important determinant of its effect on graft survival. Treatment with eclizumab has been associated with good prophylactic and therapeutic outcomes post-transplant APS-related TMA, as it inhibits complement at the C5 level.^[1,12–14]^

In conclusion, we present 3 recent cases of renal transplant recipients with APS. We designed anticoagulation therapy protocols to be applied in the perioperative period to prevent graft thrombosis and bleeding complications by lowering the target aPTT or INR. Graft function was successfully maintained within the normal range in all 3 patients, although the follow-up periods are relatively short (Fig. 2). Our findings indicate that if an accurate diagnosis in made and the appropriate anticoagulation therapy is administered in the perioperative period, living donor renal transplantation can be performed successfully in patients with APS. Nonetheless, long-term graft outcomes needed to be evaluated by examining more cases with long-term follow-up.
